# Effects of transcranial direct current stimulation on non-motor functions in individuals with Parkinson’s disease: a systematic review and meta-analysis

**DOI:** 10.3389/fnins.2025.1713623

**Published:** 2025-12-17

**Authors:** Qiyu Li, Hanmei Ye, Cheng Ye, Mi Huang

**Affiliations:** 1 Chongqing Hospital of Jiangsu Province Hospital, Chongqing, China; 2 The Affiliated Yongchuan Hospital of Chongqing Medical University, Chongqing, China; 3 Bolin Central Health Center, Chongqing, China

**Keywords:** Parkinson’s disease, tDCS, cognition, non-motor symptom, meta-analysis

## Abstract

**Background:**

Parkinson’s disease (PD) is a highly prevalent neurodegenerative disorder that poses a significant mental and physical burden on patients, markedly diminishing their quality of life. This study aimed to systematically evaluate the effects of transcranial direct current stimulation (tDCS) on cognitive functions, mood state, sleep function, and overal quality of life in individuals with PD.

**Methods:**

Relevant literature was sourced from multiple databases, ultimately including 22 studies with a total of 1324 individuals. Data analysis was conducted using meta-analysis software. The protocol was registered in PROSPERO (CRD42024599943).

**Results:**

The findings indicated that tDCS significantly enhanced cognitive function, alleviated anxiety and depression, increased sleep duration, improved sleep efficiency, reduced arousal index, and ameliorated daytime sleepiness, while also enhancing activities of daily living. However, discrepancies were noted across various assessment scales, including the Mini-Mental State Examination, the Parkinson’s Disease Cognitive Rating Scale, delayed recall metrics, sleep scales, and Parkinson’s Disease Questionnaire. Additionally, individuals with PD displayed good tolerance to tDCS.

**Conclusion:**

Overall, tDCS shows promise in improving non-motor symptoms and enhancing quality of life for individuals with PD. Nonetheless, large-scale trials are necessary to confirm these results further.

**Systematic review registration:**

https://www.crd.york.ac.uk/PROSPERO/view/CRD42024599943, Identifier: CRD42024599943.

## Introduction

Parkinson’s disease (PD) is a progressive neurodegenerative disorder primarily characterized by motor symptoms such as tremors, rigidity, bradykinesia, and postural instability. However, PD also presents significant challenges beyond motor dysfunction, with cognitive decline, mood disturbances, and impaired sleep quality further diminishing the quality of life for affected individuals (Hayes, 2019). The pathophysiology of PD involves the degeneration of dopaminergic neurons in the substantia nigra, leading to a cascade of neurobiological changes that affect various neurotransmitter systems (Cuenca et al., 2018). As the disease progresses, patients often experience a decline in cognitive functions, with 80% of individuals with PD may develop some form of cognitive impairment, including dementia (Pigott et al., 2015; Arie et al., 2017). These non-motor symptoms significantly impacts daily functioning and quality of life, necessitating effective therapeutic interventions.

Current treatments for PD primarily focus on alleviating motor symptoms through pharmacological approaches, particularly dopaminergic therapies. However, these treatments often fall short in addressing non-motor symptoms and may lead to complications such as motor fluctuations and dyskinesias over time (Bloem et al., 2015; Bryant et al., 2011). Consequently, there is a growing interest in non-invasive neuromodulation techniques, such as transcranial direct current stimulation (tDCS), as adjunct therapies that may enhance cognitive function, mental health, and improve overall well-being in patients and health adults (De Smet et al., 2024; Nasim et al., 2024; Giustiniani et al., 2024).

tDCS is a form of brain stimulation that involves the application of a low electrical current to the scalp via electrodes. This technique is believed to modulate neuronal excitability and synaptic plasticity, potentially leading to improvements in motor and non-motor symptoms (Chase et al., 2019). Several studies have reported positive outcomes associated with tDCS in various neurological and psychiatric conditions, including depression, stroke, and traumatic brain injury (Chen et al., 2024; Verma et al., 2024). In the context of PD, preliminary researches suggest that tDCS may enhance cognitive performance, particularly in areas such as attention, memory, and executive function (Pol et al., 2021; Gu et al., 2018). Furthermore, tDCS has been shown to have mood-enhancing effects, which could be particularly beneficial in addressing the high prevalence of depression and anxiety among individuals with PD (Manenti et al., 2018; Hadoush et al., 2021).

Despite these promising findings, the effects of tDCS on non-motor symptoms, such as cognition, mood, sleep function, and quality of life, remain inadequately understood. Significant heterogeneity in study designs, stimulation parameters, and outcome measures across the current literature has led to inconsistent conclusions. Notably, several systematic reviews and meta-analyses have focused on the effects of tDCS on motor function in PD, while comprehensive evaluations specifically targeting non-motor symptoms remain relatively scarce. Given the increasing recognition of the clinical importance of non-motor symptoms and the therapeutic potential tDCS has demonstrated in this domain, this review aims to elucidate the effects of tDCS on cognitive function, mood state, sleep function, and quality of life in individuals with PD, thereby informing clinical practice and directing future researches efforts.

## Methods

### Data sources and searches

This meta-analysis was conducted in accordance with the Preferred Reporting Items for Systematic Reviews and Meta-Analyses (PRISMA) guidelines (Page et al., 2021). A comprehensive literature search was performed across several databases, including Wanfang, China National Knowledge Infrastructure (CNKI), China Science and Technology Journal Database (VIP), China Biology Medicine (CBM), PubMed, Cochrane Database, Web of Science, and Embase, covering articles published from the inception of these databases until May15, 2025. The search strategy employed a combination of keywords and controlled vocabulary terms related to transcranial direct current stimulation, Parkinson’s disease and Randomized Controlled Trials. The details of the search strategy (PubMed as an example) were provided in Supplementary material. Following the removal of duplicate records, the titles and abstracts of the remaining citations were screened for potential inclusion. Full-text articles were then thoroughly reviewed to determine eligibility based on predefined criteria. The protocol for this meta-analysis has been registered in PROSPERO under the registration number CRD42024599943.

### Eligibility criteria

Articles were selected based on the PICOS framework. (1) Participants: Individuals diagnosed with Parkinson’s disease. (2) Interventions: Experimental groups received tDCS interventions either alone or in combination with rehabilitation therapies, including physical therapy and cognitive training. (3) Comparisons: Control groups were subjected to therapies without tDCS or received sham tDCS. (4) Outcomes: The primary outcome measures focused on cognitive function, while secondary outcomes included mood state, sleep quality, and overall quality of life. (5) Study Design: Only randomized controlled trials (RCTs) were included in this meta-analysis.

### Outcome measures

As the primary outcome, cognitive function was assessed using three standardized tools: the Montreal Cognitive Assessment (MoCA), the Mini-Mental State Examination (MMSE), and the Parkinson’s Disease Cognitive Rating Scale (PD-CSR). Additionally, we conducted a statistical analysis of seven cognitive domains, including visuospatial and executive function, language, attention, orientation, abstraction, naming, and delayed recall.

Secondary outcomes included mood state, sleep function, and quality of life. The mood state was evaluated using the Beck Depression Inventory (BDI), the Self-Rating Depression Scale (SDS), the Hamilton Depression Scale (HAMD), and the Hamilton Anxiety Scale (HAMA). Sleep function was assessed by measuring total sleep time, sleep efficiency, arousal index, somnolence scale, and sleep scale. Quality of life was evaluated using the Activities of Daily Living (ADL) scale, the 39-item Parkinson’s Disease Questionnaire (PDQ-39), and the 8-item Parkinson’s Disease Questionnaire (PDQ-8).

The acceptability of the intervention and the incidence of adverse events were also examined. Acceptability was measured by the number of participants who withdrew from the study for any reason throughout the intervention period. The incidence of adverse events was determined based on the number of uncomfortable symptoms reported by participants during and after the intervention.

### Data extraction and quality assessment

Two independent authors (QL and HY) conducted data extraction and assessed the risk of bias. In cases of disagreement, a third author (CY) was consulted to reach a resolution. The extracted data included study characteristics, participant characteristics, intervention details, and outcome measures. The risk of bias for the included studies was assessed using the Cochrane Risk of Bias tool, which evaluated the following domains: random sequence generation, allocation concealment, blinding of participants and personnel, blinding of outcome assessment, incomplete outcome data, selective reporting, and other potential biases.

### Data analysis

The assessment of changes in non-motor symptoms was conducted by evaluating the mean change in the respective outcome measures. In cases where the reported data included median and interquartile range, 95% confidence intervals (CIs), or means and standard errors, estimates were recalculated to provide the mean and standard deviation.

Data analysis was performed using RevMan5.3 and Stata17.0 software. Heterogeneity among studies was assessed using the Q statistic and the I^2^ statistic. For continuous outcomes, effect sizes were expressed as standardized mean differences (SMD) or mean differences (MD), accompanied by their corresponding95% CIs. Dichotomous outcomes were evaluated using risk ratios (RR) with95% CIs. A fixed-effects model was applied when heterogeneity was low (*I*^2^ ≤ 50%); conversely, a random-effects model was utilized for all analyses when significant heterogeneity was observed (*I*^2^ > 50%). Statistical significance was established at *p* < 0.05.

Begg’s and Egger’s tests were performed to assess the presence of publication bias, with *p* > 0.05 indicating no evidence of publication bias. Sensitivity analysis was performed by systematically excluding each study to evaluate the robustness of the results.

## Results

**Figure 1:**
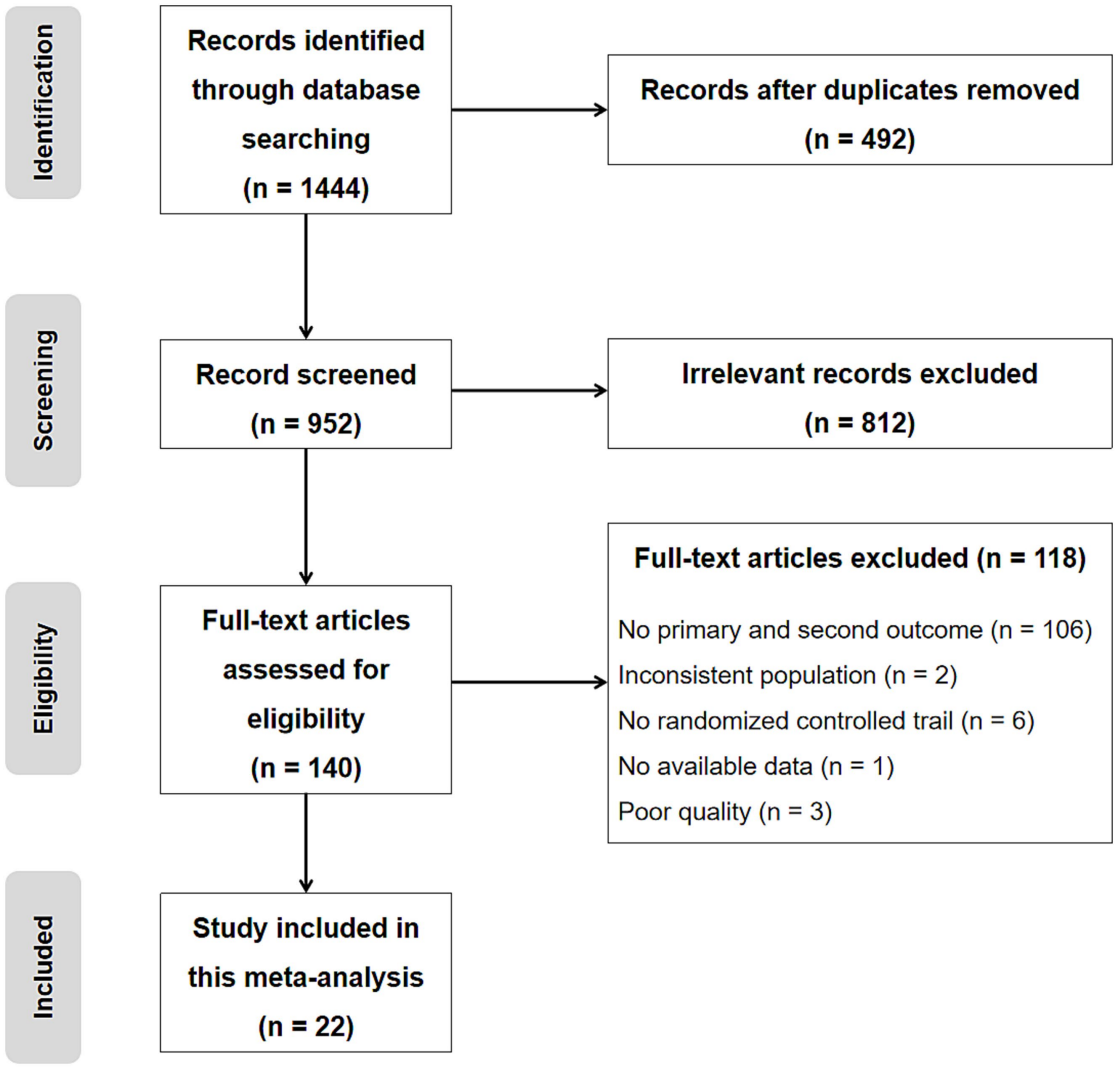
Study flowchart.

A total of 1,444 articles were retrieved from the databases. Following screening and application of the eligibility criteria, 22 studies (Aksu et al., 2022; Benninger et al., 2010; Lawrence et al., 2018; Manenti et al., 2016; Manenti et al., 2018; Pisano et al., 2024; Wong et al., 2024; Jing et al., 2022; Dong-hao et al., 2022; Xilian et al., 2021; Xue et al., 2018; Donghui and Dahua, 2021; Jianjun et al., 2024; Li et al., 2020; Chao et al., 2022; Dongchuan et al., 2016; Shaopu et al., 2020; Shaopu et al., 2023; Yang and Zhou, 2023; Jing et al., 2020; Dacheng, 2020; Zhu, 2020) were included in the meta-analysis. Figure 1 provides a flowchart of the search results, detailing the reasons for excluding specific studies.

A total of 1,324 participants (666 participants in the experimental group and 658 participants in the control group) were included in the meta-analysis. Among the included studies, the intervention sessions varied from 8 to 60. The most commonly targeted area for tDCS stimulation was the dorsolateral prefrontal cortex (DLPFC), followed by the motor cortex and the cerebellum. The stimulation intensity was set at 1–2 mA, with a single stimulation duration of 10–20 min. The main clinical and demographic characteristics of the included studies are summarized in Table 1.

**Table 1:** Overview of included studies in the meta-analysis.

Study	Number of participants	Age (mean)	H&Y stage	Intervention method	Stimulation parameters	Treatment sessions	Electrode position	Outcome indicator
	E / C	E / C		E / C			Anodal / Cathodal	
Aksu et al. (2022)	13 / 13	65.52 / 65.52	1–3	tDCS / sham tDCS	2 mA 20 min	10	Left DLPFC / right DLPFC	Semantic fluency score, Wechsler Memory Scale
Benninger et al. (2010)	13 / 12	63.60 / 64.20	2–4	tDCS / sham tDCS	2 mA 20 min	8	Alternation between the motor and prefrontal cortex / mammillary body	TST, BDI
Jing et al. (2022)	63 / 63	62.23 / 62.40	1–5	tDCS + rehabilitation / rehabilitation	2 mA 25 min	NM	DLPFC contralateral to the more PD-affected side / contralateral supraorbital area	MMSE, MoCA, BI
Dong-hao et al. (2022)	30 / 30	68.16 / 68.34	1–4	tDCS + rehabilitation / rehabilitation	2 mA 20 min	20	Left DLPFC / contralateral supraorbital area	MoCA, visuospatial and executive function, language, attention, delayed recall, orientation, abstraction, naming, BI
Xilian et al. (2021)	49 / 49	64.23 / 63.68	1–5	tDCS + rehabilitation / rehabilitation	2 mA 25 min	42	DLPFC contralateral to the more PD-affected side / contralateral supraorbital area	MMSE, MoCA
Lawrence et al. (2018)	14 / 14	65.50 / 66.86	NM	tDCS + rehabilitation / rehabilitation	1.5 mA 20 min	12	Left DLPFC /contralateral supraorbital area	MMSE, PD-CSR, COWAT, Letter-Number Sequencing, paragraph recall, BNT, PDQ-39
Xue et al. (2018)	28 / 28	64.32 / 64.39	1–2	tDCS / sham tDCS	2 mA 20 min	56	Cz / supraorbital area	MoCA, visuospatial and executive function, language, attention, delayed recall, orientation, abstraction, naming
Donghui and Dahua (2021)	30 / 30	59.70 / 60.20	≤3	tDCS + rehabilitation / rehabilitation	1.4 mA 20 min	20	Alternation between the left and right cerebellum / contralateral shoulder	BI
Jianjun et al. (2024)	29 / 28	58.00 / 59.00	NM	tDCS + medicine / medicine	2 mA 20 min	14	Left DLPFC / right DLPFC	Functional Independence Measure, TST, SE
Manenti et al. (2016)	10 / 10	69.00 / 69.10	1–3	tDCS + rehabilitation / rehabilitation	2 mA 25 min	10	Left or right DLPFC / contralateral supraorbital area	MMSE, PD-CSR, FAB, TMT-A, Naming Objects of IPNP, PDQ-39, RBDSQ
Manenti et al. (2018)	11 / 11	65.50 / 63.80	≤3	tDCS + rehabilitation / rehabilitation	2 mA 25 min	10	Left DLPFC / contralateral supraorbital area	PD-CSR, FAB, Verbal Fluency, TMT-A, Rey Auditory Verbal Learning Test, PDQ-39, RBDSQ, BDI
Pisano et al. (2024)	9 / 8	71.00 / 65.30	2–3	tDCS + rehabilitation / rehabilitation	2 mA 20 min	10	Cerebellum / right arm	MMSE, MoCA, Index of Independence in ADL, PDQ-8, BDI
Li et al. (2020)	11 / 11	62.00 / 65.00	≤3	tDCS + rehabilitation / rehabilitation	2 mA 20 min	20	Left DLPFC / contralateral supraorbital area	MMSE, MoCA
Chao et al. (2022)	43 / 42	64.41 / 63.96	1–5	tDCS + rehabilitation / rehabilitation	2 mA 25 min	60	DLPFC contralateral to the more PD-affected side / contralateral supraorbital area	MMSE, MoCA, SDS, SAS
Wong et al. (2024)	17 / 17	68.10 / 66.80	1–3	tDCS + rehabilitation / rehabilitation	2 mA 20 min	12	Left DLPFC / contralateral supraorbital area	PDQ-39
Dongchuan et al. (2016)	50 / 50	60.40 / 59.70	NM	tDCS + medicine / medicine	1 mA 10 min	28	left DLPFC / contralateral supraorbital area	BI
Shaopu et al. (2020)	28 / 26	61.00 / 62.60	≤4	tDCS + medicine / medicine	1.2 mA 20 min	20	Bilateral DLPFC / shoulder	PDQ-39, TST, SE, AI, ESS, PDSS, HAMD
Shaopu et al. (2023)	30 / 30	59.70 / 56.53	1–3	tDCS + rehabilitation / rehabilitation	2 mA 20 min	20	Left DLPFC / right DLPFC	MoCA, visuospatial and executive function, language, attention, delayed recall, orientation, abstraction, naming
Yang and Zhou (2023)	46 / 45	72.17 / 71.84	1–3	tDCS + medicine / medicine	2 mA 20 min	20	Left DLPFC / right DLPFC	HAMA, HAMD
Jing et al. (2020)	35 / 34	58.63 / 58.64	NM	tDCS + medicine / medicine	1.2 mA 20 min	30	Bilateral DLPFC / shoulder	TST, SE, AI, ESS, PSQI
Dacheng (2020)	72 / 72	61.30 / 61.30	NM	tDCS + medicine / medicine	1 mA 10 min	56	Left DLPFC / right DLPFC	MoCA, visuospatial and executive function, language, attention, delayed recall, orientation, abstraction, naming
Zhu (2020)	35 / 35	77.06 / 77.11	NM	tDCS / sham tDCS	2 mA 20 min	24	Left DLPFC /contralateral supraorbital area	MMSE, MoCA

tDCS, transcranial direct current stimulation; NM, not mentioned; DLPFC, dorsolateral prefrontal cortex; TST, total sleep time; BDI, the beck depression inventory; MMSE, the mini-mental state examination; MoCA, the montreal cognitive assessment; BI, barthel index; PD-CSR, the Parkinson’s Disease Cognitive Rating Scale; COWAT, controlled oral word association test; BNT, boston naming test; PDQ-39, the 39-item parkinson’s disease questionnaire; SE, sleep efficiency; FAB, frontal assessment battery; TMT-A, trial making test-A; RBQSQ, REM sleep behavior disorder screening questionnaire; ADL, activities of daily living; PDQ-8, the 8-item parkinson’s disease questionnaire-mentation; SDS, Self-rating Depression Scale; SAS, Self-Rating Anxiety Scale; AI, arousal index; ESS, Epworth Sleepiness Scale; PDSS, Parkinson Disease Sleep Scale; HAMD, the Hamilton Depression Scale; HAMA, the Hamilton Anxiety Scale; PSQI, Pittsburgh sleep quality index.

### Primary outcome: cognitive function

**Figure 2:**
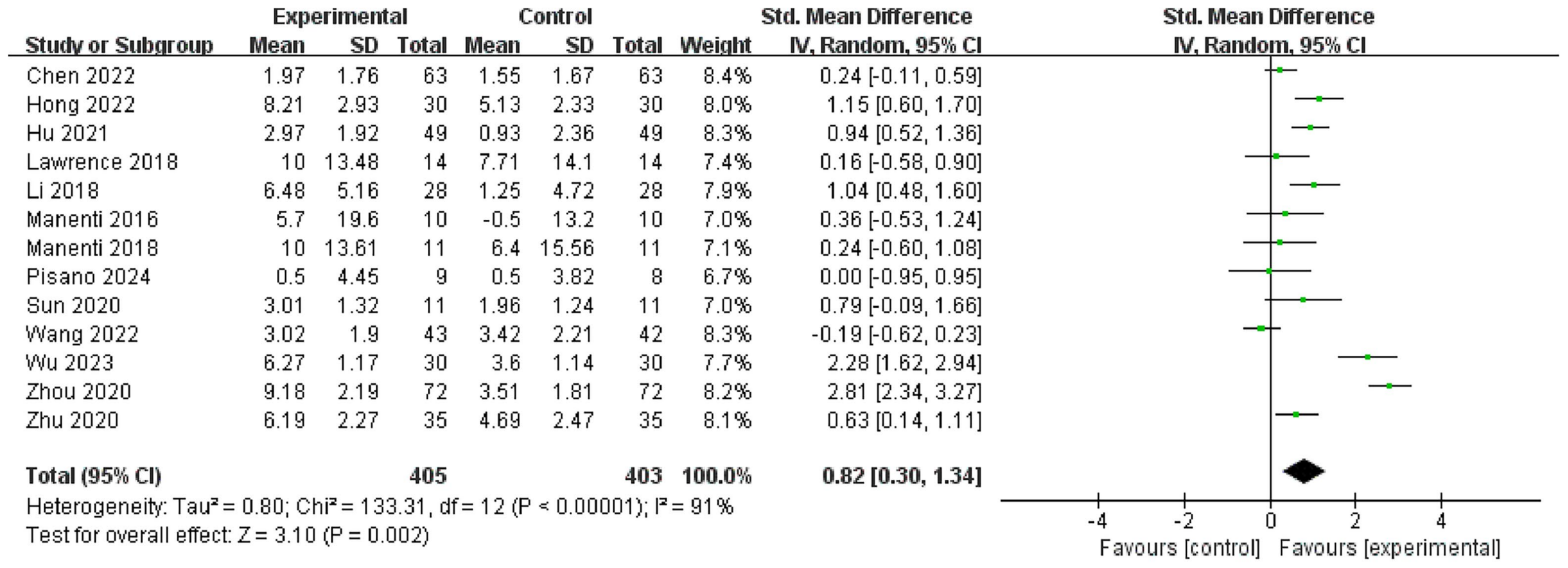
Forest plot. Cognitive function.

The meta-analysis demonstrated a significant advantage of tDCS over control conditions in enhancing cognitive function, with a SMD of 0.82 (13 studies, 95% CI 0.30 to 1.34, *Z* = 3.10, *p* = 0.002) and high heterogeneity (*I*^2^ = 91%) (Figure 2).

**Figure 3:**
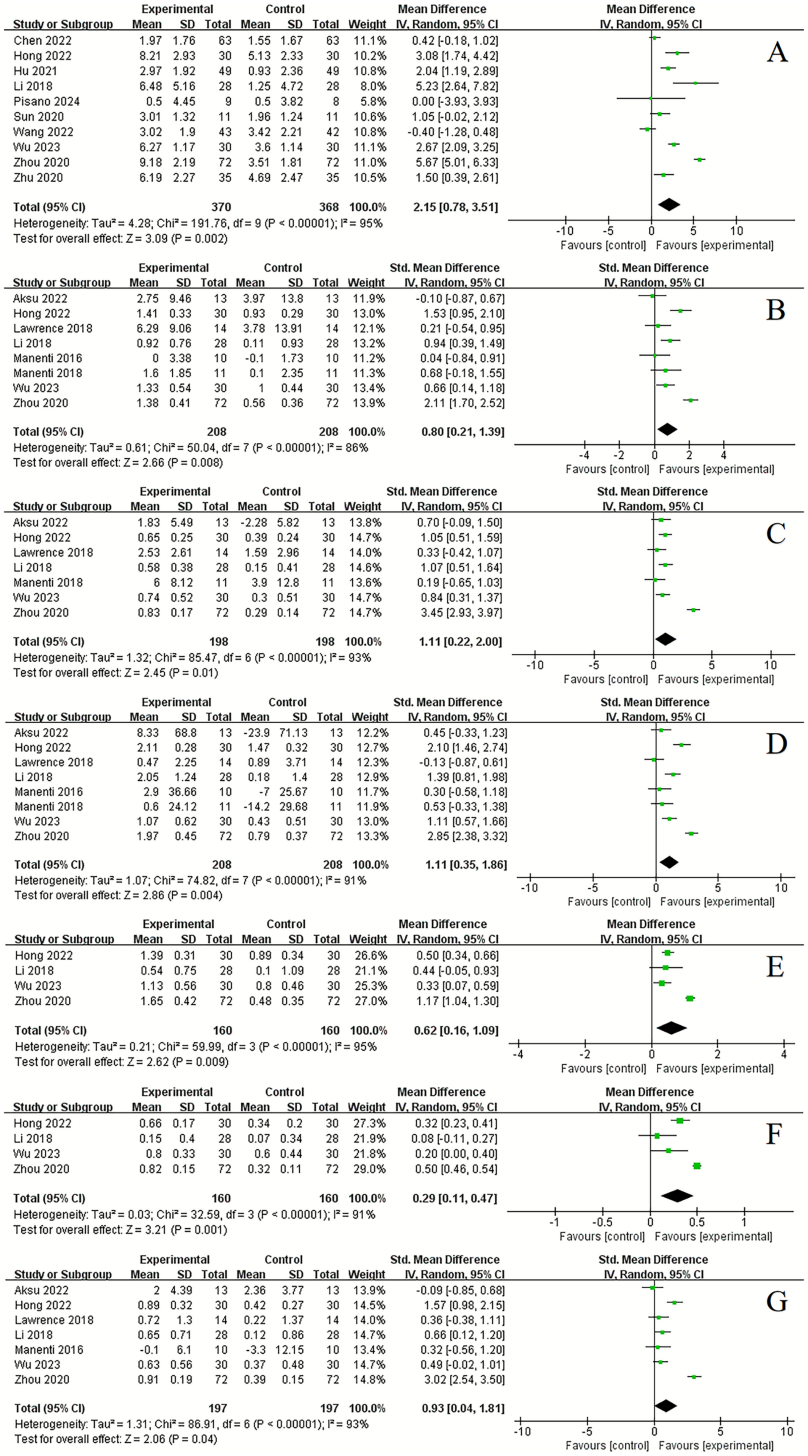
Forest plot. Effects of tDCS on cognitive function evaluated using MoCA **(A)**, visuospatial and executive function **(B)**, language **(C)**, attention **(D)**, orientation **(E)**, abstraction **(F)**, naming **(G)**.

**Figure 4:**
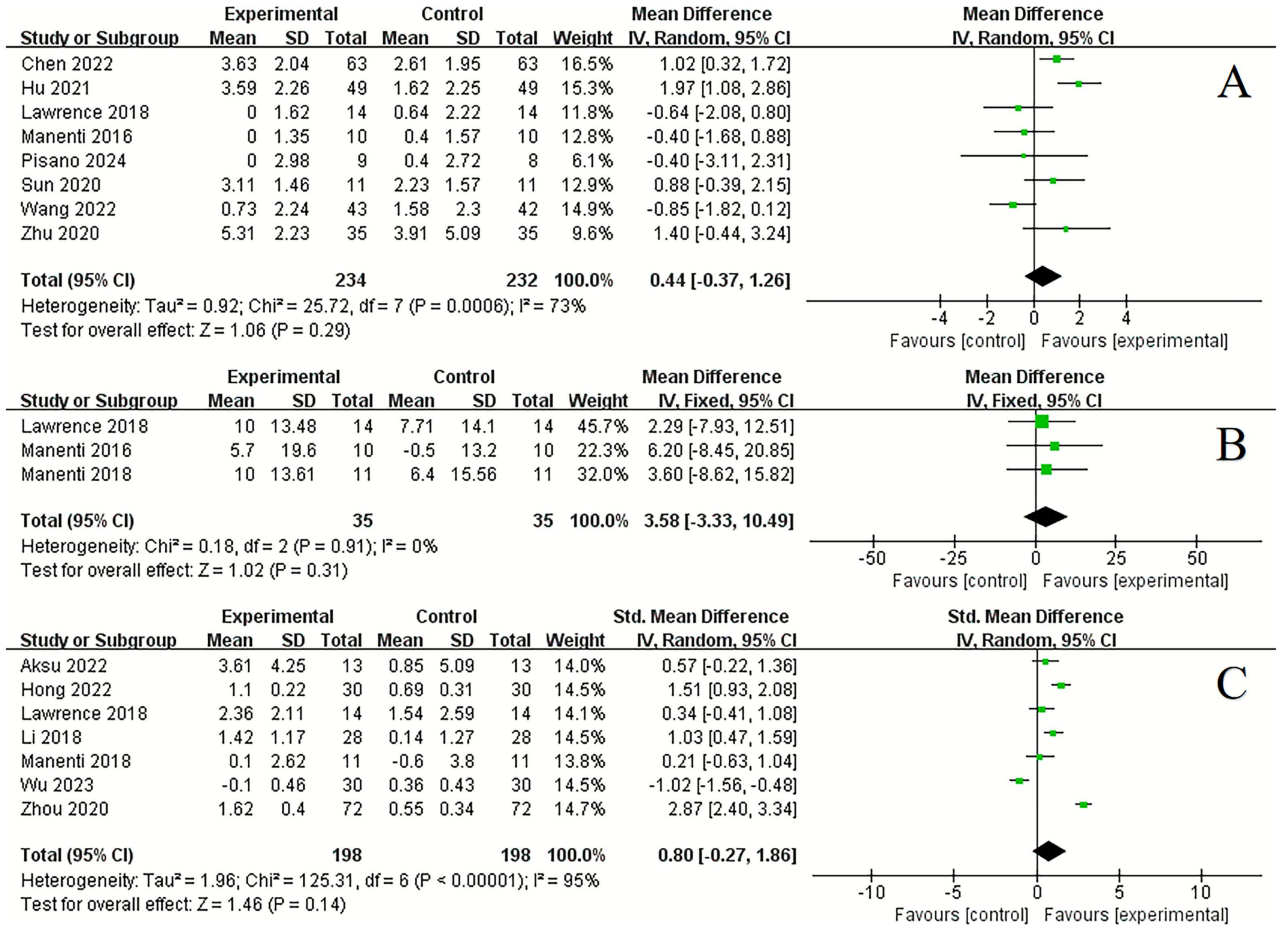
Forest plot. Effects of tDCS on cognitive function evaluated using MMSE **(A)**, PD-CSR **(B)**, delayed recall **(C)**.

Cognitive function assessment included measures such as MoCA, MMSE, PD-CSR. Additionally, we analyzed changes in various cognitive domains before and after the intervention. Significant improvements following tDCS were observed in MoCA (10 studies, MD = 2.15, 95% CI 0.78 to 3.51, *p* = 0.002, *I*^2^ = 95%) (Figure 3A), visuospatial and executive function (8 studies, SMD = 0.80, 95% CI 0.21 to 1.39, *p* = 0.008, *I*^2^ = 86%) (Figure 3B), language (7 studies, SMD = 1.11, 95% CI 0.22 to 2.00, *p* = 0.01, *I*^2^ = 93%) (Figure 3C), attention (8 studies, SMD = 1.11, 95% CI 0.35 to 1.86, *p* = 0.004, *I*^2^ = 91%) (Figure 3D), orientation (4 studies, MD = 4, 95% CI 0.62 to 1.09, *p* = 0.009, *I*^2^ = 95%) (Figure 3E), abstraction (4 studies, MD = 0.29, 95% CI 0.11 to 0.47, *p* = 0.001, *I*^2^ = 91%) (Figure 3F), and naming (7 studies, SMD = 0.93, 95% CI 0.04 to 1.81, *p* = 0.04, *I*^2^ = 93%) (Figure 3G). However, no significant improvements were found in MMSE (8 studies, MD = 0.44, 95% CI −0.37 to 1.26, *p* = 0.29, *I*^2^ = 73%) (Figure 4A), PD-CSR (3 studies, MD = 3.58, 95% CI −3.33 to 10.49, *p* = 0.31, *I*^2^ = 0%) (Figure 4B), and delayed recall (7 studies, SMD = 0.80, 95% CI −0.27 to 1.86, *p* = 0.14, *I*^2^ = 95%) (Figure 4C).

### Secondary outcomes: mood state, sleep function, and quality of life

**Figure 5:**
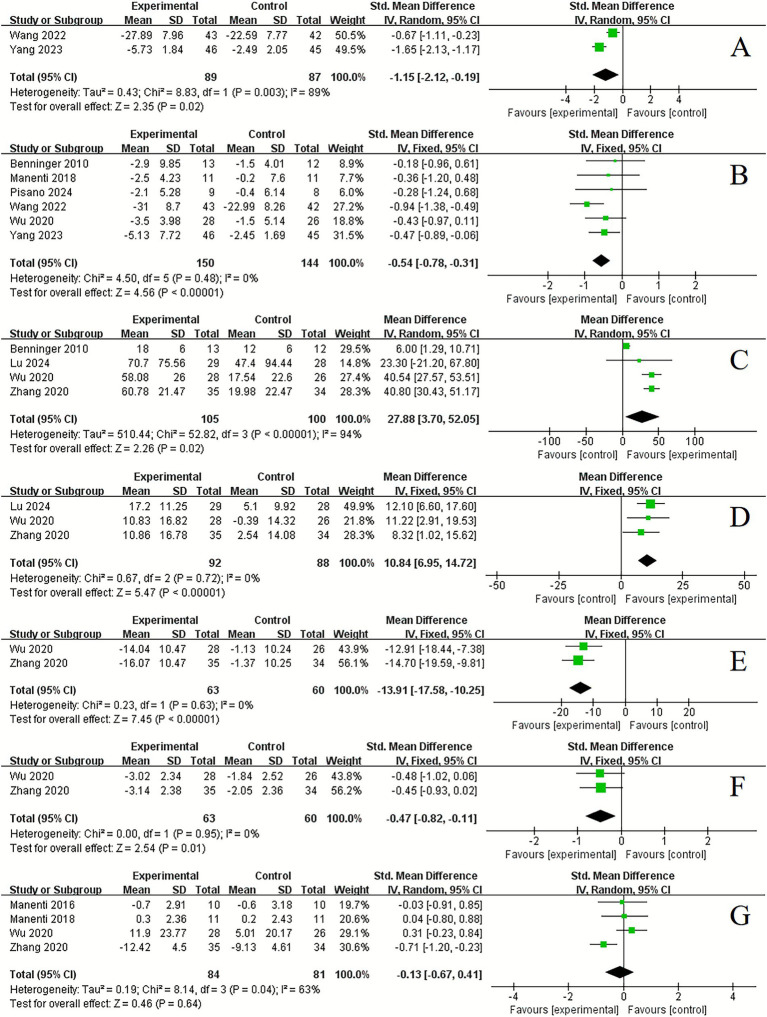
Forest plot. Effects of tDCS on mood state and sleep function evaluated using anxious **(A)**, depression **(B)**, total sleep time **(C)**, sleep efficiency **(D)**, arousal index **(E)**, somnolence scale **(F)**, sleep scale **(G)**.

In the assessment of mood state, tDCS demonstrated a significant improvement compared to control conditions in anxious (2 studies, SMD = −1.15, 95% CI −2.12 to −0.19, *p* = 0.02, *I*^2^ = 89%) (Figure 5A) and depression (6 studies, SMD = −0.54, 95% CI −0.78 to −0.31, *p* < 0.001, *I*^2^ = 0%) (Figure 5B).

Regarding sleep function, significant improvements were observed following tDCS compared to control conditions in total sleep time (4 studies, MD = 27.88, 95% CI 3.70 to 52.05, *p* = 0.02, *I*^2^ = 94%) (Figure 5C), sleep efficiency (3 studies, MD = 10.84, 95% CI 6.95 to 14.72, *p* < 0.001, *I*^2^ = 0%) (Figure 5D), arousal index (3 studies, MD = −13.91, 95% CI −17.58 to −10.25, *p* < 0.001, *I*^2^ = 0%) (Figure 5E) and somnolence scale (2 studies, SMD = −0.47, 95% CI −0.82 to −0.11, *p* = 0.01, *I*^2^ = 0%) (Figure 5F). However, no significant improvement was found in sleep scale (4 studies, SMD = −0.13, 95% CI −0.67 to 0.41, *p* = 0.64, *I*^2^ = 63%) (Figure 5G).

**Figure 6:**
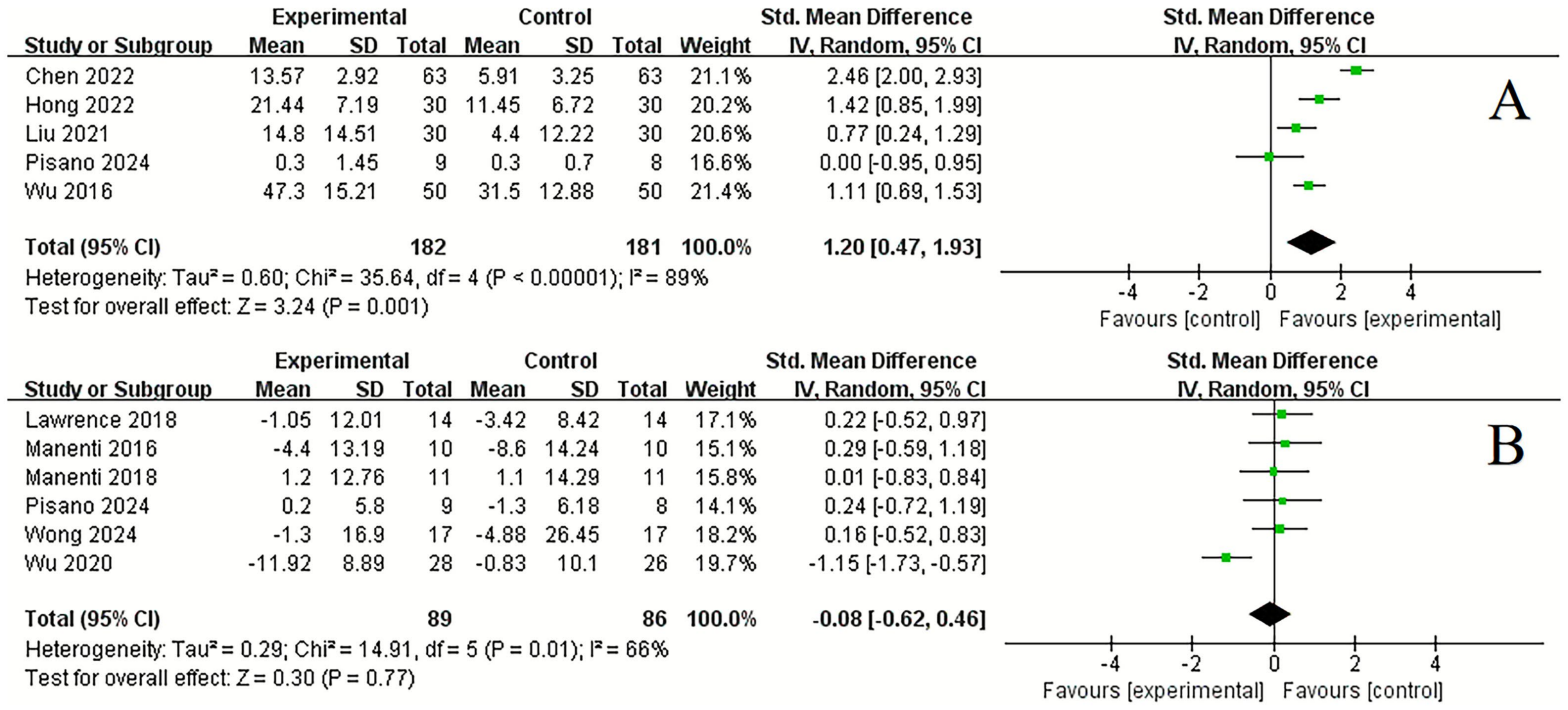
Forest plot. Effects of tDCS on quality of life evaluated using ADL **(A)**, PDQ **(B)**.

In terms of quality of life, significant improvements were noted following tDCS compared to control conditions in ADL (5 studies, SMD = 1.20, 95% CI 0.47 to 1.93, *p* = 0.001, *I*^2^ = 89%) (Figure 6A). However, there was no significant improvement in PDQ (6 studies, SMD = −0.08, 95% CI −0.62 to 0.46, *p* = 0.77, *I*^2^ = 66%) (Figure 6B).

### Dropout rate and adverse events

In the studies analyzed, dropout rates were observed in both the experimental and control groups. Specifically, in the experimental group, three participants (0.45%) withdrew from the study, while in the control group, two participants (0.30%) opted out. The comparison of dropout rates between the two groups revealed no significant difference (RR = 1.48, 95% CI 0.23 to 9.68, *p* = 0.68). Notably, the reasons for withdrawal were not related to the intervention itself. Throughout the course of the studies, no adverse reactions were reported.

### Sensitivity analyses and reporting bias

**Figure 7:**
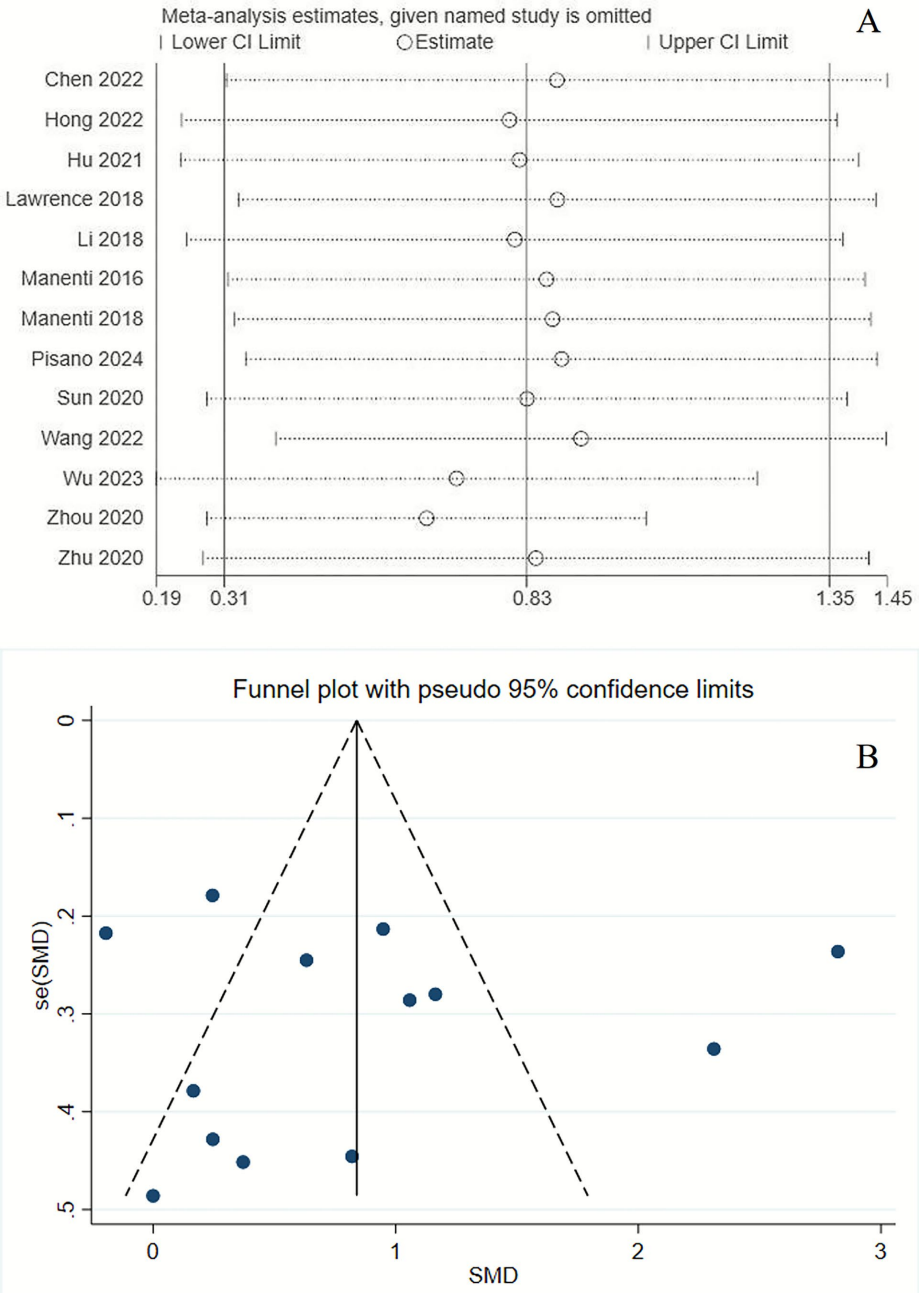
Sensitivity analysis of primary outcome **(A)** and funnel chart for publication bias **(B)**.

Substantial heterogeneity was observed across studies regarding the primary outcomes (*I*^2^ = 91%). A sensitivity analysis was performed to assess the robustness of the findings related to cognitive function. The inclusion of missing studies did not alter the overall effect of tDCS on cognitive function (Figure 7A). The funnel plot indicated potential publication bias (Figure 7B). However, both Egger’s test (*p* = 0.936) and Begg’s test (*p* = 0.855) showed no significant evidence of publication bias.

### Subgroup analyses

To explore potential sources of the substantial heterogeneity in main outcomes (*I*^2^ = 91%), we conducted subgroup analyses by frequency of intervention (≤20 sessions vs. >20 sessions) and stimulation intensity (2 mA vs. <2 mA). The results showed no statistically significant differences between subgroups for either intervention frequency (*χ*^2^ = 0.25, df = 1, *p* = 0.62) or stimulation intensity (*χ*^2^ = 0.37, df = 1, *p* = 0.54).

### Quality assessment

**Figure 8:**
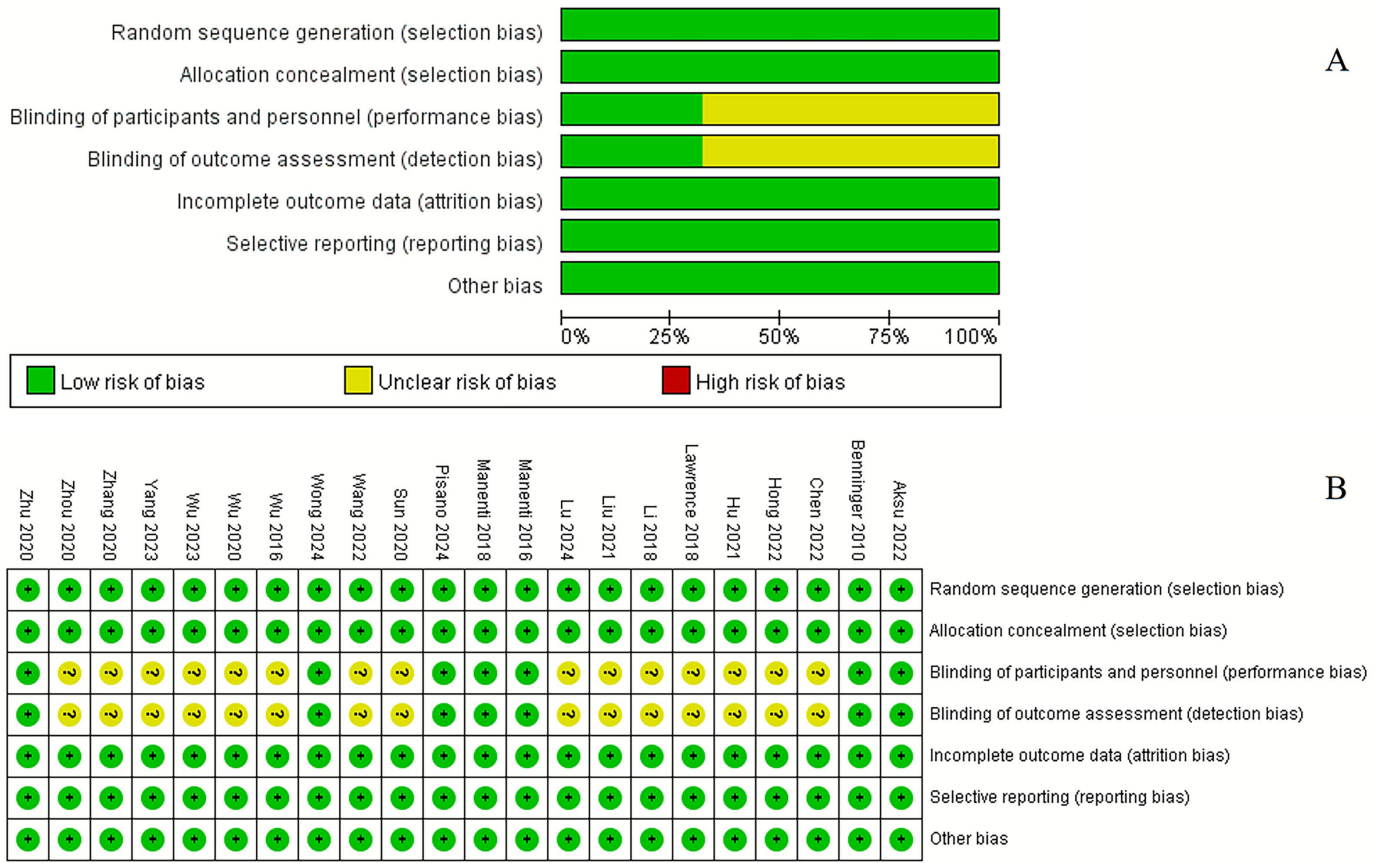
Risk of bias analysis and quality assessment of included trials. **(A)** Risk of bias graph: judgement of review authors about each risk of bias item presented as percentages across all included studies. **(B)** Risk of bias summary: judgement of review authors about each risk of bias item for each included study.

The assessment of bias risk in the included studies is illustrated in Figure 8. Overall, the quality of the studies was deemed to be moderate to high. Certain domains, such as performance bias and detection bias, were rated as having a moderate risk of bias.

## Discussion

Previous meta-analyses have explored the effects of tDCS on PD symptoms, yielding mixed conclusions. While most earlier studies have primarily focused on the improvement of motor symptoms associated with PD, they have not thoroughly examined the therapeutic effects of tDCS on non-motor symptoms, particularly concerning cognitive and sleep functions. Our study offers a comprehensive analysis of the impacts of tDCS on cognitive function, mood state, sleep quality, and overall quality of life in individuals with PD. Our findings demonstrate that tDCS significantly enhances cognitive function, mental well-being, sleep quality, and the overall quality of life for individuals with PD. Importantly, the treatment was well-tolerated, with no significant adverse effects reported.

Cognitive impairment in PD often begins subtly and progresses slowly, making it difficult to detect early. Approximately 40% of individuals with PD present with mild cognitive impairment in the early stages of the disease (Pfeiffer et al., 2013). This impairment typically presents as dysfunction in one or more cognitive domains, with deficits in visuospatial and executive functions being particularly prominent (Muslimović et al., 2005). Treatment options for cognitive impairment in PD are limited, and medications used to address motor symptoms may exacerbate cognitive deficits, particularly anticholinergic agents (Williams-Gray et al., 2013). In recent years, there has been a growing interest in non-pharmacological interventions to enhance cognitive function, including computer-assisted cognitive training and non-invasive neurostimulation techniques such as tDCS.

Our meta-analysis indicates that tDCS significantly improves cognitive function in individuals with PD. However, variations in assessment methods yielded differing results. Notable improvements were observed in MoCA scores, visuospatial and executive functions, language, attention, orientation, abstraction, and naming. In contrast, there were no significant changes in MMSE scores, PD-CSR, or delayed recall. These findings are consistent with those reported by Liu et al. (2021), which also showed that tDCS improved MoCA scores in individuals with PD, but did not affect MMSE or PD-CSR outcomes. The MMSE and MoCA are widely utilized cognitive assessment tools. However, the MMSE is primarily used for dementia screening and has lower sensitivity for detecting mild cognitive impairment. Conversely, the MoCA demonstrates higher sensitivity and specificity for mild cognitive decline (Ciesielska et al., 2016; Nasreddine et al., 2005). Given that the PD population in our included studies did not exhibit overt dementia, the MoCA results may more accurately reflect cognitive changes in individuals with PD. Furthermore, our findings suggest that tDCS exerts positive effects across various cognitive domains, aligning with results from Suarez-García et al. (2020). However, due to the limited number of studies assessing the impact of tDCS on cognitive outcomes in PD and the small sample sizes for cognitive subdomain analyses, the reliability of our findings may be somewhat limited. Future clinical researches are necessary to investigate the efficacy of tDCS in improving overall cognitive function and specific cognitive domains in individuals with PD.

Among the 12 studies included in the cognitive function assessment, two utilized stimulation targets in the cerebellum and central area, while the remainder focused on the dorsolateral prefrontal cortex (DLPFC). Current researches on the effects of tDCS on cognitive function in individuals with PD predominantly concentrate on the DLPFC. Notably, some studies have shown that tDCS targeting non-DLPFC brain regions can also enhance cognitive function (Ishikuro et al., 2018), suggesting that effective neurostimulation targets are not limited to the DLPFC. The mechanisms underlying the effects of tDCS on cognitive deficits remain unclear but may involve an increase in local cerebral blood flow and improvement of brain function (Rizzo et al., 2014). Other researches have also indicated that the therapeutic effects of tDCS are closely associated with its specific modulation of brain functional connectivity, as tDCS can promote topological reorganization of brain networks. Therefore, tDCS may ultimately improve cognitive function in individuals with PD through multiple mechanisms, including modulation of cortical inhibitory circuits, enhancement of cortical excitability, and regulation of brain neural networks and dopamine levels (Broeder et al., 2015; Simonetta et al., 2025).

Approximately 31% of individuals with PD suffer from depression, while 40% to 60% experience anxiety disorders (Huang et al., 2023). Additionally, approximately 47.66% to 89.10% of individuals with PD have sleep disturbances, primarily manifesting as excessive daytime sleepiness, rapid eye movement sleep behavior disorder, periodic leg movements during sleep, and sleep-disordered breathing (Liu et al., 2018; Falup-Pecurariu and Diaconu, 2017). Compared to motor symptoms, emotional and sleep-related issues are often overlooked, yet they significantly impact patients’ quality of life and may influence disease progression (Oliveira de Carvalho et al., 2018). The mechanisms underlying PD-related depression are associated with dopaminergic depletion, impairment of the frontostriatal circuitry, degeneration of monoaminergic neurotransmitter systems, and dysfunction of the limbic system (Cuenca et al., 2018). Additionally, the accumulation of alpha-synuclein leads to neurodegenerative changes in noradrenergic, dopaminergic, and serotoninergic neurons, resulting in neuronal cell death in the pathways that affect the thalamocortical arousal system and the brainstem’s sleep–wake control centers, thereby altering sleep architecture (Monti and Monti, 2007; Mahmood et al., 2020).

Current treatment recommendations for emotional and sleep disorders in PD primarily focus on improving the environment, engaging in exercise therapy, pharmacological interventions, and using transcranial magnetic stimulation. Our meta-analysis indicates that tDCS can significantly reduce anxiety and depression scores in individuals with PD, extend sleep duration, enhance sleep quality, and alleviate daytime sleepiness. However, the specific mechanisms through which tDCS improves emotional and sleep disturbances in individuals with PD remain incompletely understood. Possible explanation is that tDCS modulates cerebral blood flow in the stimulated cortical areas, leading to sustained and widespread changes in neuronal activity, enhanced synaptic connectivity, and regulation of monoaminergic systems within deep brain structures (Hadoush et al., 2021; Clark et al., 2011).

Collectively, these findings underscore the potential of tDCS as a multifaceted intervention for addressing both mental health and sleep issues in individuals with PD. However, the lack of significant improvement in specific sleep scales raises questions regarding the sensitivity of these measures to detect changes following tDCS. It is conceivable that while objective sleep parameters demonstrated improvement, the subjective perceptions of sleep quality may not have been adequately captured by the scales employed.

In this study, we observed significant improvements in ADL following tDCS in individuals with PD, highlighting the potential of tDCS as a beneficial intervention for enhancing quality of life in this patient population. These findings corroborate existing literature that supports the use of neuromodulation techniques to improve functional outcomes (Salazar et al., 2017; Wang et al., 2024). However, it is noteworthy that no significant changes were detected in the PDQ, which suggests that while tDCS may enhance specific functional abilities, it may not influence broader quality-of-life measures or subjective experiences reported by patients. This discrepancy prompts further investigation into the nuances of how tDCS impacts both objective and subjective assessments of quality of life in PD, and underscores the importance of utilizing a comprehensive set of evaluation tools to fully understand the therapeutic effects of tDCS in this context.

The heterogeneity observed in the analysis of primary outcomes suggests variations in effect sizes across the included studies. However, sensitivity analyses indicate that the overall effect estimate was not significantly influenced by any single study, thus reinforcing the robustness of our findings. Subgroup analyses based on the number of sessions and stimulation intensity still revealed no significant differences. Furthermore, the absence of significant publication bias implies that our overall results were not distorted by selective publication practices. The generalizability of our findings is somewhat limited by substantial heterogeneity. To enhance precision and clinical applicability in future researches, subsequent studies should focus on establishing standardized tDCS protocols and promoting individual participant data meta-analyses. Such approaches would enable a more nuanced understanding of tDCS efficacy across different subgroups of Parkinson’s disease, thereby supporting more targeted clinical guidance.

### Study limitations

Several limitations should be acknowledged in this meta-analysis. First, some of the studies included had small sample sizes, which may potentially lead to an overestimation of effect sizes. Second, there is a possibility of publication bias, as researchers are often less inclined to report negative findings. Additionally, high heterogeneity was observed in the primary outcomes. To explore the sources of this heterogeneity, we conducted sensitivity analyses and assessed bias, which demonstrated the robustness of our findings. Furthermore, many of the included studies lacked long-term follow-up data, preventing us from evaluating the sustained benefits of rhythmically cued exercise interventions. Importantly, there is a notable scarcity of research specifically targeting the improvement of non-motor symptoms in individuals with PD, and the stimulation targets in existing studies are relatively limited. These gaps highlight the need for further investigation in these critical areas.

## Conclusion

In conclusion, tDCS shows promise for improving certain non-motor symptoms in individuals with PD. Based on a systematic evaluation of 22 studies involving 1,347 participants, tDCS effectively enhances cognitive function, alleviates anxiety and depression, promotes longer and more efficient sleep, reduces arousal indices, and mitigates daytime sleepiness. Furthermore, tDCS can also improve the abilities of daily living activities. However, methodological limitations and variability in outcome measures across studies preclude definitive conclusions. Future researches should prioritize standardized assessment tools and larger-scale randomized controlled trials to better establish the efficacy and long-term benefits of tDCS in PD management.

## Data Availability

The original contributions presented in the study are included in the article/Supplementary material, further inquiries can be directed to the corresponding author.

## References

[ref1] Aksu S. Uslu A. İşçen P. Tülay E. E. Barham H. Soyata A. Z. . (2022). Does transcranial direct current stimulation enhance cognitive performance in Parkinson’s disease mild cognitive impairment? An event-related potentials and neuropsychological assessment study. Neurol. Sci. 43, 4029–4044. doi: 10.1007/s10072-022-06020-z, 35322340

[ref2] Arie L. Herman T. Shema-Shiratzky S. Giladi N. Hausdorff J. (2017). Do cognition and other non-motor symptoms decline similarly among patients with Parkinson's disease motor subtypes? Findings from a 5-year prospective study. J. Neurol. 264, 2149–2157. doi: 10.1007/s00415-017-8605-x 28879438

[ref3] Benninger D. H. Lomarev M. Lopez G. Wassermann E. M. Li X. Considine E. . (2010). Transcranial direct current stimulation for the treatment of Parkinson's disease. J. Neurol. Neurosurg. Psychiatry 81, 1105–1111. doi: 10.1136/jnnp.2009.202556, 20870863 PMC4162743

[ref4] Bloem B. R. de Vries N. M. Ebersbach G. (2015). Nonpharmacological treatments for patients with Parkinson's disease. Mov. Disord. 30, 1504–1520. doi: 10.1002/mds.26363. 26274930

[ref5] Broeder S. Nackaerts E. Heremans E. Vervoort G. Meesen R. Verheyden G. . (2015). Transcranial direct current stimulation in Parkinson's disease: neurophysiological mechanisms and behavioral effects. Neurosci. Biobehav. Rev. 57, 105–117. doi: 10.1016/j.neubiorev.2015.08.010 26297812

[ref6] Bryant M. S. Rintala D. H. Hou J. G. Lai E. C. Protas E. J. (2011). Effects of levodopa on forward and backward gait patterns in persons with Parkinson's disease. NeuroRehabilitation 29, 247–252. doi: 10.3233/NRE-2011-0700 22142758 PMC3391536

[ref7] Chao W. Dewang N. Wenbo W. Chaosheng G. (2022). The effect of transcranial direct current stimulation combined with personalized rehabilitation education on mood, cognitive function and three-dimensional gait in patients with Parkinson's disease. J. Int. Psychiatry. 49, 904–907. doi: 10.13479/j.cnki.jip.2022.05.027

[ref8] Chase H. Boudewyn M. Carter C. Phillips M. (2019). Transcranial direct current stimulation: a roadmap for research, from mechanism of action to clinical implementation. Mol. Psychiatry 25, 397–407. doi: 10.1038/s41380-019-0499-9, 31455860 PMC6981019

[ref9] Chen Y. Zhao Z. Huang J. Wang T. Qu Y. (2024). Computer-aided cognitive training combined with tDCS can improve post-stroke cognitive impairment and cerebral vasomotor function: a randomized controlled trial. BMC Neurol. 24:132. doi: 10.1186/s12883-024-03613-3, 38641827 PMC11027365

[ref10] Ciesielska N. Sokołowski R. Mazur E. Podhorecka M. Polak-Szabela A. Kędziora-Kornatowska K. (2016). Is the Montreal cognitive assessment (MoCA) test better suited than the Mini-mental state examination (MMSE) in mild cognitive impairment (MCI) detection among people aged over 60? Meta-analysis. Psychiatr. Pol. 50, 1039–1052. doi: 10.12740/PP/45368, 27992895

[ref11] Clark V. Coffman B. Trumbo M. Gasparovic C. (2011). Transcranial direct current stimulation (tDCS) produces localized and specific alterations in neurochemistry: a 1H magnetic resonance spectroscopy study. Neurosci. Lett. 500, 67–71. doi: 10.1016/j.neulet.2011.05.244, 21683766

[ref12] Cuenca L. Gil-Martinez A. L. Cano-Fernandez L. Sanchez-Rodrigo C. Estrada C. Fernandez-Villalba E. . (2018). Parkinson’s disease: a short story of 200 years. Histol. Histopathol. 34, 573–591. doi: 10.14670/HH-18-073, 30540129

[ref13] Dacheng Z. (2020). The effects of selegiline tablets combined with transcranial direct current stimulation on the cognitive function of patients with Parkinson's disease. Mod. Pract. Med. 32, 1057–1059. doi: 10.3969/j.issn.1671-0800.2020.09.015

[ref14] De Smet S. Razza L. Pulopulos M. de R. Baeken C. Brunoni A. . (2024). Stress priming transcranial direct current stimulation (tDCS) enhances updating of emotional content in working memory. Brain Stimul. 17, 434–443. doi: 10.1016/j.brs.2024.03.021 38565374

[ref15] Dongchuan W. Xiaoxia W. Guangjun W. Zhenhua Z. Jian L. Zhongai K. . (2016). Effect of oral dopa hydrazine tablets combined with transcranial direct current stimulation on non-motor symptoms in patients with Parkinson’s disease. Shandong Pharmaceut. 56, 88–90. doi: 10.3969/j.issn.1002-266X.2016.31.029

[ref16] Dong-hao H. Shan-yao Z. Hai-yan C. Min X. Xiao-fei Y. Wei-min Y. (2022). Effects of rehabilitation exercise training combined with transcranial direct current stimulation on walking function, balance function and cognitive function in patients with Parkinson's disease. Prog. Mod. Biomed. 22, 2575–2578. doi: 10.13241/j.cnki.pmb.2022.13.034

[ref17] Donghui L. Dahua Z. (2021). Observation of the efficacy of transcranial direct current stimulation in improving balance disorders in patients with Parkinson's disease. Chin. J. Med. 56, 765–768. doi: 10.3969/j.issn.1008-1070.2021.07.021

[ref18] Falup-Pecurariu C. Diaconu Ş. (2017). Sleep dysfunction in Parkinson’s disease. Int. Rev. Neurobiol. 133, 719–742. doi: 10.1016/bs.irn.2017.05.033, 28802939

[ref19] Giustiniani A. Maistrello L. Mologni V. Danesin L. Burgio F. (2024). TMS and tDCS as potential tools for the treatment of cognitive deficits in Parkinson's disease: a meta-analysis. Neurol. Sci. 46, 579–592. doi: 10.1007/s10072-024-07778-0 39320648

[ref20] Gu B. Wang Q. Lei X. Song L. (2018). Advance in transcranial direct current stimulation for cognitive impairment in Parkinson's disease. Chin. J. Rehabil. Theory Pract. 24, 773–778. doi: 10.3969/j.issn.1006-9771.2018.07.005

[ref21] Hadoush H. Alqudah A. Banihani S. al-Jarrah M. Amro A. Aldajah S. (2021). Melatonin serum level, sleep functions, and depression level after bilateral anodal transcranial direct current stimulation in patients with Parkinson's disease: a feasibility study. Sleep Sci 14, 25–30. doi: 10.5935/1984-0063.20200083, 34917270 PMC8663735

[ref22] Hayes M. T. (2019). Parkinson’s disease and parkinsonism. Am. J. Med. 132, 802–807. doi: 10.1016/j.amjmed.2019.03.001, 30890425

[ref23] Huang X. Dong K. Gan C. Xu Z. Lei D. Dong X. . (2023). Effect of rhythmically cued exercise interventions on functions in patients with Parkinson disease: a meta-analysis. Phys. Ther. 104:pzad158. doi: 10.1093/ptj/pzad158, 37962936

[ref24] Ishikuro K. Dougu N. Nukui T. Yamamoto M. Nakatsuji Y. Kuroda S. . (2018). Effects of transcranial direct current stimulation (tDCS) over the frontal polar area on motor and executive functions in Parkinson's disease; a pilot study. Front. Aging Neurosci. 10:231. doi: 10.3389/fnagi.2018.00231 30104971 PMC6077209

[ref25] Jianjun L. Yu H. Qiumin Y. Jiawen L. Minghua Z. Jinzhi L. . (2024). Effects of transcranial direct current stimulation on sleep disorders in Parkinson’s disease:a randomized, single - blind controlled trial. J. Pract. Med. 40, 1488–1493. doi: 10.3969/j.issn.1006-5725.2024.11.004

[ref26] Jing C. Binglong L. Wengang T. (2022). Effect of transcranial direct current stimulation combined with routine rehabilitation training on Parkinson disease. Chin. J. Pract. Med. 49, 56–59. doi: 10.3760/cma.j.cnl15689-20220718-03136

[ref27] Jing Z. Xiaoyun W. Junnan W. Jie J. Dong L. Weiping A. (2020). Effects of transcranial direct current stimulation on cognitive and neurological functions in patients with Parkinson’s disease with REM sleep behavior disorder. Prog. Mod. Biomed. 20, 4182–4185. doi: 10.13241/j.cnki.pmb.2020.21.041

[ref28] Lawrence B. J. Gasson N. Johnson A. R. Booth L. Loftus A. M. (2018). Cognitive training and transcranial direct current stimulation for mild cognitive impairment in Parkinson's disease: a randomized controlled trial. Parkinsons Dis. 2018, 1–12. doi: 10.1155/2018/4318475, 29780572 PMC5892209

[ref29] Li S. Shu W. Wei Y. Dan M. (2020). Clinical efficacy of transcranial direct current stimulation combined with cognitive training in the improvement of cognitive impairment in Parkinson disease. Chin. J. Rehabil. 35, 308–311.

[ref30] Liu X. Liu H. Liu Z. Rao J. Wang J. Wang P. . (2021). Transcranial direct current stimulation for Parkinson’s disease: a systematic review and meta-analysis. Front. Aging Neurosci. 13:746797. doi: 10.3389/fnagi.2021.746797 34776931 PMC8584149

[ref31] Liu C. F. Wang T. Zhan S. Q. Geng D. Q. Wang J. Liu J. . (2018). Management recommendations on sleep disturbance of patients with Parkinson's disease. Chin. Med. J. 131, 2976–2985. doi: 10.4103/0366-6999.247210 30539911 PMC6302643

[ref32] Mahmood Z. van R. Nakhla M. Twamley E. Filoteo J. Schiehser D. (2020). REM sleep behavior disorder in Parkinson’s disease: effects on cognitive, psychiatric, and functional outcomes. J. Int. Neuropsychol. Soc. 26, 894–905. doi: 10.1017/S1355617720000430, 32375913 PMC7554050

[ref33] Manenti R. Brambilla M. Benussi A. Rosini S. Cobelli C. Ferrari C. . (2016). Mild cognitive impairment in Parkinson’s disease is improved by transcranial direct current stimulation combined with physical therapy. Mov. Disord. 31, 715–724. doi: 10.1002/mds.26561, 26880536

[ref34] Manenti R. Cotelli M. Cobelli C. Gobbi E. Brambilla M. Rusich D. . (2018). Transcranial direct current stimulation combined with cognitive training for the treatment of Parkinson disease: a randomized, placebo-controlled study. Brain Stimul. 11, 1251–1262. doi: 10.1016/j.brs.2018.07.046, 30056141

[ref35] Monti J. M. Monti D. (2007). The involvement of dopamine in the modulation of sleep and waking. Sleep Med. Rev. 11, 113–133. doi: 10.1016/j.smrv.2006.08.003, 17275369

[ref36] Muslimović D. Post B. Speelman J. Schmand B. (2005). Cognitive profile of patients with newly diagnosed Parkinson disease. Neurology 65, 1239–1245. doi: 10.1212/01.wnl.0000180516.69442.95, 16247051

[ref37] Nasim Z. Sajjad B. Hamidreza G. L. Mohammad N. Michael A. N. Mohammad Ali S. (2024). Repeated prefrontal tDCS for improving mental health and cognitive deficits in multiple sclerosis: a randomized, double-blind, parallel-group study. J. Transl. Med. 22:843. doi: 10.1186/s12967-024-05638-1, 39272101 PMC11397099

[ref38] Nasreddine Z. S. Phillips N. A. Bédirian V. Charbonneau S. Whitehead V. Collin I. . (2005). The Montreal cognitive assessment, MoCA: a brief screening tool for mild cognitive impairment. J. Am. Geriatr. Soc. 53, 695–699. doi: 10.1111/j.1532-5415.2005.53221.x 15817019

[ref39] Oliveira de Carvalho A. ASS F. Murillo-Rodriguez E. Rocha N. B. Carta M. G. Machado S. (2018). Physical exercise for Parkinson's disease: clinical and experimental evidence. Clin. Pract. Epidemiol. Ment. Health 14, 89–98. doi: 10.2174/1745017901814010089 29785199 PMC5897963

[ref40] Page M. J. JE M. K. Bossuyt P. M. Boutron I. Hoffmann T. C. Mulrow C. D. . (2021). The PRISMA 2020 statement: an updated guideline for reporting systematic reviews. BMJ 372:n71. doi: 10.1136/bmj.n71 33782057 PMC8005924

[ref41] Pfeiffer H. C. Løkkegaard A. Zoetmulder M. Friberg L. Werdelin L. (2013). Cognitive impairment in early-stage non-demented Parkinson's disease patients. Acta Neurol. Scand. 129, 307–318. doi: 10.1111/ane.12189 24117192

[ref42] Pigott K. Rick J. Xie S. X. Hurtig H. Chen-Plotkin A. Duda J. E. . (2015). Longitudinal study of normal cognition in Parkinson disease. Neurology 85, 1276–1282. doi: 10.1212/WNL.0000000000002001, 26362285 PMC4617168

[ref43] Pisano F. Mellace D. Fugatti A. Aiello E. N. Diotti S. Curti B. . (2024). Cerebellar tDCS combined with augmented reality treadmill for freezing of gait in Parkinson's disease: a randomized controlled trial. J. Neuroeng. Rehabil. 21:173. doi: 10.1186/s12984-024-01457-z, 39342307 PMC11438075

[ref44] Pol F. Salehinejad M. A. Baharlouei H. Nitsche M. A. (2021). The effects of transcranial direct current stimulation on gait in patients with Parkinson's disease: a systematic review. Transl. Neurodegener. 10:22. doi: 10.1186/s40035-021-00245-2 34183062 PMC8240267

[ref45] Rizzo V. Terranova C. Crupi D. Sant'angelo A. Girlanda P. Quartarone A. (2014). Increased transcranial direct current stimulation after effects during concurrent peripheral electrical nerve stimulation. Brain Stimul. 7, 113–121. doi: 10.1016/j.brs.2013.10.002, 24388283

[ref46] Salazar A. P. S. Vaz P. G. Marchese R. R. Stein C. Pinto C. Pagnussat A. S. (2017). Noninvasive brain stimulation improves hemispatial neglect after stroke: a systematic review and meta-analysis. Arch. Phys. Med. Rehabil. 99, 355–366.e1. doi: 10.1016/j.apmr.2017.07.009, 28802812

[ref47] Shaopu W. Xue L. Yawei Q. Heng W. Jianjun M. (2020). The influence of transcranial direct current stimulation on rapid eye movement sleep disorders among persons with Parkinson’s disease. Chin. J. Phys. Med. Rehabil. 42, 50–54. doi: 10.3760/cma.jissn.0254-1424.2020.01.012

[ref48] Shaopu W. Xue L. Yawei Q. Heng W. Jianjun M. (2023). Exercising to music combined with transcranial direct current stimulation improves the motor and cognitive functioning of persons with Parkinson′s disease. Chin. J. Phys. Med. Rehabil. 45, 678–682. doi: 10.3760/cma.j.issn.0254-1424.2023.08.002

[ref49] Simonetta C. Conti M. Bissacco J. Ferrari V. Salimei C. Carparelli F. . (2025). Anodal M1 tDCS shapes frequency-specific functional connectivity and network topology in Parkinson's disease. Brain Stimul. 18, 1966–1977. doi: 10.1016/j.brs.2025.10.016, 41151703

[ref50] Suarez-García D. M. A. Grisales-Cárdenas J. S. Zimerman M. Cardona J. F. (2020). Transcranial direct current stimulation to enhance cognitive impairment in Parkinson's disease: a systematic review and meta-analysis. Front. Neurol. 11:597955. doi: 10.3389/fneur.2020.597955 33329353 PMC7734248

[ref51] Verma R. Ganesh R. Narnoli S. Nandakumar D. Sharma P. Sharma K. . (2024). Effectiveness and tolerability of adjunctive transcranial direct current stimulation (tDCS) in management of treatment-resistant depression: a retrospective chart review. Indian J. Psychiatry 66, 538–544. doi: 10.4103/indianjpsychiatry.indianjpsychiatry_243_24 39100375 PMC11293779

[ref52] Wang Y. Ding Y. Guo C. (2024). Assessment of noninvasive brain stimulation interventions in Parkinson’s disease: a systematic review and network meta-analysis. Sci. Rep. 14:14219. doi: 10.1038/s41598-024-64196-0, 38902308 PMC11189909

[ref53] Williams-Gray C. Mason S. Evans J. Foltynie T. Brayne C. Robbins T. . (2013). The campaign study of Parkinson's disease: 10-year outlook in an incident population-based cohort. J. Neurol. Neurosurg. Psychiatry 84, 1258–1264. doi: 10.1136/jnnp-2013-305277, 23781007

[ref54] Wong P. L. Yang Y. R. Huang S. F. Wang R. Y. (2024). Effects of DLPFC tDCS followed by treadmill training on dual-task gait and cortical excitability in Parkinson's disease: A randomized controlled trial. Neurorehabil. Neural Repair 38, 680–692. doi: 10.1177/15459683241268583, 39104216

[ref55] Xilian H. Cuiping X. Zishuang L. (2021). The effects of transcranial direct current stimulation-assisted functional rehabilitation training on the recovery of patients with Parkinson's disease. Chin. J. Gerontol. 41, 3724–3727. doi: 10.3969/j.issn.1005-9202.2021.17.027

[ref56] Xue L. Junhong Z. Yawei Q. Shaopu W. Hongyan D. Hongqi Y. . (2018). Effects of transcranial direct current stimulation on cognitive function and auditory event-related potentials in patients with Parkinson’s disease. Chin. J. Phys. Med. Rehabil. 40, 198–201. doi: 10.3760/cma.j.issn.0254-1424.2018.03.009

[ref57] Yang H.-n. Zhou S.-j. (2023). Effects of transcranial direct current stimulation on motor function, psychological state, and serum neurotransmitters in elderly patients with Parkinson's disease. Sichuan J. Physiol. Sci. 45, 244–247.

[ref58] Zhu W. (2020). Clinical observation of transcranial direct current stimulation combined with pramipexole in the treatment of Parkinson’s disease. Tibetan Med. 41, 73–74.

