# Supplementary material for: Effects of transcranial direct current stimulation on non-motor functions in individuals with Parkinson’s disease: a systematic review and meta-analysis
Source: Front Neurosci. 2025 Dec 17;19:1713623. doi: 10.3389/fnins.2025.1713623 (PMC12753959; doi:10.3389/fnins.2025.1713623)
Supplement: Supplementary file 1 [file Table_1.docx]

**Search strategy in PubMed**

#1 Search “Parkinson disease” [MeSH]

#2 Search (Parkinson’s disease[Title/Abstract])

#3 Search “transcranial direct current stimulation” [MeSH]

#4 Search (((((((tDCS[Title/Abstract])) AND (Transcranial Electrical Stimulation[Title/Abstract])) AND (Electrical Stimulations, Transcranial[Title/Abstract])) AND (Electrical Stimulation, Transcranial[Title/Abstract])) AND (Stimulations, Transcranial Electrical[Title/Abstract])) AND (Stimulation, Transcranial Electrical[Title/Abstract])) AND (Transcranial Electrical Stimulations[Title/Abstract])))))))

#5 Search “Randomized Controlled Trials” [MeSH]

#6 Search ((((Clinical Trials, Randomized[Title/Abstract]) OR (Trials, Randomized Clinical[Title/Abstract]) OR (Controlled Clinical Trials, Randomized[Title/Abstract]) OR (RCT[Title/Abstract]))))

#7 #1 OR #2

#8 #3 OR #4

#9 #5 OR #6

#10 #7 AND #8 AND # 9
